# Natural *vs*. artificial cycle for frozen embryo transfer: impact on clinical and obstetric outcomes

**DOI:** 10.5935/1518-0557.20260045

**Published:** 2026

**Authors:** Maria do Carmo Borges de Souza, Roberto de Azevedo Antunes, Marcelo Marinho de Souza, Ana Cristina Allemand Mancebo, Thaísa Damasceno Renovato, Verônica de Almeida Raupp, Ana Luísa Bruno Marinho de Souza, Flávia Fernandes Sequeira, Brenda Maria Loureiro de Melo, Karina Abelha Rabaço

**Affiliations:** 1 FERTIPRAXIS Clinic - Human Reproduction Center, Rio de Janeiro, Brazil; 2 Department of Gynecology, Clementino Fraga Filho University Hospital, UFRJ - UFRJ - Federal University of Rio de Janeiro, Rio de Janeiro, Brazil

**Keywords:** natural cycle, artificial cycle, FET, progesterone support

## Abstract

**Objective:**

To compare clinical, obstetric, and neonatal outcomes between natural and artificial endometrial preparation protocols for frozen embryo transfer (FET) in cycles involving single euploid blastocyst transfer.

**Methods:**

This retrospective observational study included only single embryo transfers of thawed day 5 or 6 good-quality blastocysts submitted to preimplantation genetic testing for aneuploidy (PGT-A) between January 2022 and May 2024. Patients were allocated to either natural or artificial endometrial preparation groups, and each group was further stratified into three subgroups according to luteal phase support: oral dydrogesterone, vaginal micronized progesterone, or combined treatment. Exclusion criteria were submucosal fibroids, endometrial polyps, intramural fibroids ≥5 cm, and hydrosalpinx detected on transvaginal ultrasound. Clinical pregnancy, live birth, miscarriage, neonatal birth weight, and gestational complications were analyzed using odds ratios (OR), confidence intervals (CI), *p*-values, generalized linear models, and z test, as appropriate.

**Results:**

The natural cycle group showed significantly better reproductive outcomes, with higher clinical pregnancy rates (OR=0.37; 95%CI: 0.13-1.02; *p*=0.047) and live birth rates (OR=0.46; 95%CI: 0.24-0.89; *p*=0.022), whereas the artificial cycle group had a significantly higher miscarriage rate (OR=2.96; 95%CI: 1.51-5.96; *p*=0.002). Analysis across the six subgroups demonstrated a significant advantage in clinical pregnancy among patients undergoing natural cycles with oral progesterone support. In contrast, miscarriage was significantly more frequent in the artificial cycle subgroup receiving oral progesterone than in the corresponding natural cycle subgroup. Neonatal birth weight was higher in artificial preparation cycles by a mean of 135.81 g, although this difference was not statistically significant (95%CI: -138.29 to 409.90; *p*=0.326). Among gestational complications, only gestational diabetes differed significantly between groups (z=2.156; *p*=0.03), while no significant differences were observed for preeclampsia or HELLP syndrome.

**Conclusion:**

True natural cycles were associated with significantly higher clinical pregnancy and live birth rates, as well as lower miscarriage rates, than artificial cycles in euploid frozen embryo transfer cycles.

## INTRODUCTION

The widespread adoption of freeze-all protocols in IVF cycles has led to a progressive shift in clinical practice towards the preferential use of frozen embryo transfers (FET).

Global data presented at the 2024 ESHRE Annual Meeting demonstrated that, in 2020, 76.7% of all single blastocyst transfers involved frozen embryos (Adamson *et al*., 2024). Similarly, the 2021 Latin American Registry of Assisted Reproduction reported that 52.7% of cycles employed a freeze-all approach, with 72.5% of transfers being performed as FET (Zegers-Hochschild *et al*., 2025).

The optimal protocol for endometrial preparation in FET cycles remains an ongoing debate. The precise physiological synchrony between the euploid embryo and the endometrium represents a major focus of current research. The choice of including a corpus luteum (CL) appears to influence maternal health and may have implications for neonatal outcomes throughout pregnancy (Lawrenz *et al*., 2020), although this remains an area of active discussion (Pinborg *et al*., 2023; Ho *et al*., 2024).

The primary objective of this study was to evaluate the effectiveness of different types of endometrial preparation-natural versus artificial cycles-in the context of routine clinical practice, without the restrictive framework of a randomized controlled trial (Horwitz *et al*., 1990). As a secondary objective, we sought to compare approaches to luteal phase support and examine their association with maternal complications.

## MATERIAL AND METHODS

Retrospective, observational, real-life cohort study, just single euploid embryo transfers (SET) of thawed day 5 or 6 blastocysts previously frozen using vitrification, conducted between January 2022 and May 2024. Embryos resulted with/without the use of donor oocytes.

Patient data were obtained through review of medical records, following informed consent, at FERTIPRAXIS Clinic - Human Reproduction Center, Rio de Janeiro, Brazil, an institution accredited by the Latin American Network of Assisted Reproduction. The study protocol received approval from the Ethics Committee of Maternidade Escola, Federal University of Rio de Janeiro (UFRJ), and was registered with Plataforma Brasil under protocol number 83384024.3.0000.5275.

The choice of endometrial preparation-natural or artificial cycle-was determined by the attending physician. The decision regarding LPS was either shared with the patient or made solely by the physician, particularly in cases involving combined progesterone regimens.

### Exclusion Criteria

Submucosal fibroids, endometrial polyps, intramural or serosal fibroids ≥5 cm, or hydrosalpinx as identified by TVU, as well as an endometrial thickness < 7mm, or no confirmation of the presence of a CL in the natural cycle.

### Endometrial Preparation Protocols

#### a) True Natural Cycle (tNC):

Patients with regular menstrual cycles (21-35 days) underwent a baseline transvaginal ultrasound (TVU) by cycle day 5 to exclude residual follicles, followed by a control TVU between days 8 and 10 to confirm the presence of a dominant follicle (≥14 mm) and endometrial thickening. From the identification of the dominant follicle onward, patients performed urinary LH peak detection tests every 12 hours (Clearblue®, SPD Swiss Precision Diagnostics GmbH) notifying their physician upon obtaining a positive result, which was considerate day LH + 0 regardless of the time of the test positive. The presence of a CL was confirmed by TVU two days later (day LH + 2), as well as an endometrial thickness of ≥7mm, and luteal phase support (LPS) started on that very same day. Embryo transfer was performed 5 days later (day LH +7).

To avoid transfers on Sundays, LPS could be started on day LH+1, if endometrial thickness was at least 7mm, in which case, CL confirmation was still performed on day LH+2, but in that case, ET was performed on day LH+6.

#### b) Artificial Cycle (AC):

Endometrial preparation commenced on cycle day 2 or 3 with oral estradiol valerate at a dose of 4 mg/day (Primogyna® - Delpharm Lille S.A.S., France) or estradiol (Natifa® - Libbs Farmacêutica, Brazil). A baseline transvaginal ultrasound (TVU) was performed, and endometrial thickness was monitored between days 8 and 10 of estrogen administration. Endometrial thickness ≥7 mm was required and if this threshold was not achieved after 25 days of estrogen use, the cycle was cancelled (Stormlund *et al*., 2025). Upon reaching the required endometrial thickness, the estradiol dose was increased to 6 mg/day, and LPS was initiated (designated as day P+0). Embryo transfer was subsequently scheduled on the fifth day of progesterone administration (day P+5).

## EMBRYO THAWING AND TRANSFER

Previously frozen day 5 or 6 blastocysts were morphologically graded (Gardner & Schoolcraft, 1999); grades 4, 5, or 6, and A/or B, prior to cryopreservation and thawed following standard protocols (Ingamed®). Embryos were cultured in CSSNMXC® medium for a minimum of two hours prior to transfer.

Transfers were conducted in an ambulatory operating room under positive pressure, guided by transabdominal ultrasound using a Wallace® 17G catheter, carefully placing the embryo at the mid-point of the endometrial cavity.

### Luteal Phase Support

Three different regimens were utilized, maintained until the 12th week of gestation:

a) Micronized vaginal progesterone (MVP) [Junno® - Farmoquímica AS, Brazil; or Utrogestan® - Besins, Brazil: 200 mg every 8 hours.
b) Dydrogesterone (DYG) [Duphaston® 10 mg, Abbott Laboratórios, Brazil; 1 tablet orally every 8 hours.
c) Combination MVP (200 mg every 12 hours) + DYG (10 mg orally every 8 hours).

### Outcome Measures

Biochemical pregnancy was assessed via serum β-hCG testing 12 days post-transfer. Clinical pregnancy was defined as the presence of a gestational sac with embryonic cardiac activity on TVU, performed 15 days after a positive β-hCG result. Miscarriage was defined as the spontaneous loss of a clinical pregnancy before 22 completed weeks of gestation. Live birth rate (LBR) was defined as the number of deliveries resulting in at least one live-born infant per embryo transfer, according to international standards (Zegers-Hochschild *et al*., 2017).

### Statistical Analysis

They were performed using R software (version 4.3.2, 2023). Initially, descriptive analyses were conducted for the following variables: age, body mass index (BMI), embryo stage, neonatal birth weight (BW), and the presence of endometriosis, stratified by endometrial preparation type (natural *vs*. artificial) and their respective subgroups. Progesterone subgroups included: natural-combined, natural-vaginal, natural-oral, artificial-combined, artificial-vaginal, and artificial-oral cycles.

Quantitative variables (age, BMI, and BW) were summarized as means with standard deviations and compared using t-tests and ANOVAS. Categorical variables were presented as frequencies and percentages, with comparisons performed using Chi-square test. A *p*-value < 0.05 was considered indicative of statistical significance. Additionally, descriptive analyses of birth rates were performed across all groups.

Generalized linear models (GLM) were subsequently employed to assess associations between the study groups and the following outcomes: live birth, biochemical pregnancy, clinical pregnancy, miscarriage, and neonatal birth weight. To ensure the robustness and reliability of the findings, all models were adjusted for key potential confounders: maternal age, BMI, embryo morphology, and the presence of endometriosis.

For binary outcomes (LBR, pregnancy, clinical pregnancy, and miscarriage), results are presented as odds ratios (ORs) with 95% confidence intervals (CIs). For the continuous outcome of neonatal birth weight, results are expressed as estimated coefficients (β) with corresponding 95% CIs.

Additionally, outcome weighting was applied across groups to correct for potential imbalances in sample sizes, ensuring a more accurate estimation of group effects within the overall cohort. Weights were calculated based on the total sample size and the relative number of participants in each group.

## RESULTS

A total of 301 SET was included in the analysis. Of these, 202 were performed following a natural cycle (NC) protocol and 99 followed an artificial cycle (AC) protocol. Within the NC group, 15 cycles used combined progesterone support, 141 used vaginal, and 46 used oral progesterone. In the AC group, 26 used combined, 53 vaginal, and 20 oral progesterone.

Baseline characteristics of patients in the NC and AC groups are presented in Table 1, and subgroup comparisons are shown in Table 2. No statistically significant differences were observed between groups regarding age, BMI, neonatal birth weight and presence of endometriosis. A significant difference was noted in embryo stage distribution between groups (*p*=0.016).

**Table 1:** Sample Characteristics by Main Groups

0,358 pt	Category	Natural cycle	Artificial cycle	p value
Age		38.4 (3.34)	39.0 (3.48)	0.559
BMI		24.5 (23.8)	24.9 (23.4)	0.117
Birth weight		3150 (465)	3306 (437)	0.143
Embryo stage	4	12 (4.0)	-	**0.016**
	5	162 (54.2)	76 (25.4)	
	6	28 (9.4)	21 (7.0)	
Endometriosis		13 (4.4)	11 (3.7)	0.151

Continuous data (age, BMI, and neonatal weight) are expressed as mean ± standard deviation (t-tests).

Categorical data are expressed as frequencies and percentages (chi-square test).

The *p*-value in bold indicates a statistically significant difference between natural and artificial cycles

**Table 2:** Sample Characteristics by Subgroups

Variable	Category	Natural			Artificial			p value
		Combined	Vaginal	Oral	Combined	Vaginal	Oral	
Age		38.3 (4.35)	38.6 (3.27)	38.2 (3.27)	39.0 (3.04)	38.9 (3.62)	39.2 (3.75)	0.836
BMI		24.4 (3.40)	25.0 (4.11)	23.3 (3.39)	24.1 (3.85)	25.2 (5.95)	25.5 (4.17)	0.175
Birth weight		3255 (372)	3203 (474)	3000 (455)	3338 (556)	3278 (451)	3320 (266)	0.404
Embryo stage	4	-	4 (1.3)	8 (2.7)	-	-	-	**0.003**
	5	15 (5.0)	114 (38.1)	33 (11.0)	22 (7.4)	39 (13.0)	15 (5.0)	
	6	-	23 (7.7)	5 (1.7)	2 (0.7)	14 (4.7)	5 (1.7)	
Endometriosis	Yes	1 (0.3)	10 (3.4)	2 (0.7)	4 (1.4)	6 (2.0)	1 (0.3)	0.479

Note: Continuous data (age. BMI. and neonatal weight) are expressed as mean ± standard deviation (ANOVA).

Categorical data are expressed as frequencies and percentages (chi-square test).

The *p*-value in bold indicates a statistically significant difference between the groups

Live birth rates (LBR) by main group and subgroups are summarized in Table 3. The overall LBR was higher in the NC group (45.54%) than in the AC group (34.34%), although this difference did not reach statistical significance (z=1.85; *p*=0.06). Among subgroups, the highest LBR was observed in the natural-oral group (58.69%). No statistically significant differences were identified in subgroup comparisons.

**Table 3:** Live birth rates

Cycle	n	Number of births	Rate (%)	z (p)
Natural Artificial	202 99	92 34	45.54 34.34	1.85 (0.06)
Natural-combined Artificial-combined	15 26	8 11	53.33 42.30	0.68 (0.49)
Natural-vaginal Artificial-vaginal	141 53	57 15	40.42 28.3	1.55 (0.12)
Natural-oral Artificial-oral	46 20	27 8	58.69 40	1.39 (0.16)

Note: z=z test; *p*=statistical significance.

Results of the GLMs for the primary and secondary outcomes by main group are shown in Table 4. After adjustment for age, BMI, embryo stage, and presence of endometriosis, clinical pregnancy was significantly more likely in the NC group compared to the AC group (OR=0.37; 95% CI: 0.13-1.02; *p*=0.047). Miscarriage was significantly more frequent in the AC group (OR=2.96; 95% CI: 1.51-5.96; *p*=0.002). No significant differences were found in birth or neonatal birth weight outcomes.

**Table 4:** Results of Generalized Linear Models (GLM) Analyses (two groups).

Dependent variable	Category	Estimative (95% CI)	p
Birth	Natural^R^ Artificial	0.47 (0.18 - 1.25)	0.133
Pregnancy (B hCG+)	Natural^R^ Artificial	0.88 (0.60 - 1.30)	0.550
Clinical pregnancy	Natural^R^ Artificial	0.37 (0.13 - 1.02)	**0.047**
Abortion	Natural^R^ Artificial	2.96 (1.51 - 5.96)	**0.002**
Birth weight	Natural^R^ Artificial	135.81 (-138.29 - 409.90)	0.326
Birth weight (37-39 week)	Natural^R^ Artificial	71.86 (-240.01 - 383.74)	0.652
Birth weight (>39 week)	Natural^R^ Artificial	168.54 (-167.96 - 505.05)	0.326

Note: CI = confidence interval; p = statistical significance; R = reference values.

p-values are adjusted using the Holm method. All analyses were controlled for age, BMI, embryonic stage, and presence of endometriosis.

Subgroup analyses are reported in Table 5. Pregnancy was more likely in patients using oral progesterone in a natural cycle when compared to those using vaginal progesterone in a natural cycle (OR=2.35; 95% CI: 1.06-5.21; *p*=0.03). In addition, patients in the natural-oral group had significantly higher pregnancy rates compared to those in the artificial-oral group (OR=0.87; 95% CI: 0.22-0.93; *p*=0.036). Miscarriage was more frequent in the artificial-oral group compared to the natural-oral group (OR=1.31; 95% CI: 1.20-1.95; *p*=0.001). Full interand intra-group comparisons are presented in Supplementary Tables 1-3.

**Table 5:** Results of Generalized Linear Models (GLM) Analyses (six groups)

Dependent variable	Category	Estimative	p
Pregnancy (B-hCG)	Natural-vaginal^R^ - natural-oral	2.35 (1.06 - 5.21)	0.03
Pregnancy (B-hCG)	Natural-oral^R^ - artificial-oral	0.87 (0.22 - 0.93)	0.036
Abortion	Natural-oral^R^ - artificial-oral	1.31 (1.20 - 1.95)	0.001

Note: CI=confidence interval; *p*=statistical significance; R=reference values.

*p*-values are adjusted using the Holm method. All analyses were controlled for age. BMI. embryonic stage. and presence of endometriosis.

Clinical complications during pregnancy are presented in Table 6. Gestational diabetes occurred significantly more often in the AC group (17.6%) compared to the NC group (5.4%) (z=2.156; *p*=0.03). No statistically significant differences were observed between groups for preeclampsia or HELLP syndrome.

**Table 6:** Pregnancy complications in the general groups

Clinical diseases in pregnancy	Natural cycle n (%)	Artificial cycle n (%)	z (p)
Gestational diabetes	5 (5.4)	6 (17.6)	**2.156 (0.03)**
Preeclampsia	4 (4.3)	3 (8.8)	0.972 (0.33)
HELLP syndrome	0 (-)	1 (2.9)	1.62 (0.10)

Note: z=z test; *p*=statistical significance.

## DISCUSSION

Successful implantation requires synchrony between a viable embryo and a receptive endometrium, within the window of implantation (WOI)-a period that has been studied since the 1970s and is thought to occur between days 19 and 23 of the menstrual cycle (Psychoyos, 1973). In this retrospective cohort study, we aimed to isolate the impact of endometrial preparation protocol by limiting the analysis to euploid SET, thereby controlling for embryonic competence.

Deferring embryo transfer through frozen-thawed cycles may offer practical advantages, including improved patient autonomy and the ability to align treatment with personal or professional schedules (Alonso-Mayo *et al*., 2024). AC protocols are often perceived as ideal in this context due to reduced monitoring and predictability (Mackens *et al*., 2017; 2023). Although modified natural cycles (mNC) using ovulation induction or hCG trigger are common, our study focused exclusively on tNC, defined by spontaneous ovulation and reliance on the physiological corpus luteum, without pre-ovulatory hormonal support. This approach reflects current interest in more physiological endometrial preparation strategies (Lawrenz *et al*., 2020).

Mackens *et al*. (2023) demonstrated that the addition of just 30 mg of DYG per day to MVP could improve reproductive outcomes in women with low serum progesterone levels on the day of FET in an HRT cycle. This additional progesterone supplementation was previously supported by evidence from Gaggiotti-Marre *et al*. (2019) as a potential ‘rescue’ for HRT-FET cycles with low serum progesterone on the day prior to FET; however, they had added 25 mg/day of subcutaneous progesterone to MVP. Therefore, we decided to evaluate three options: 30 mg of DYG alone, 600 mg of MVP alone, or a combination of 400 mg MVP plus 30 mg DYG.

Criticisms of tNC often cite its dependence on precise monitoring and reduced scheduling flexibility (Reljič & Knez, 2018). However, in our setting, patients undergoing tNC required only 3-4 clinic visits. The protocol was facilitated by urinary LH testing positivity and CL confirmation via ultrasound, which allowed for scheduled initiation of progesterone support and avoided weekend transfers-consistent with the approach described by Gavrić Lovrec *et al*. (2022). No serial hormonal monitoring was necessary, in line with earlier findings on WOI timing precision using LH-based scheduling (Xiao *et al*., 2012).

Cancellation rates were comparable between groups: 15.1% in tNC (mostly due to anovulation or premature ovulation) and 14.6% in AC (primarily due to undetected ovulation or insufficient endometrial thickness). These rates are notably lower than those reported by Ho *et al*. (2024), who described a 21% cancellation rate in mNC due to premature ovulation or anovulation. Additional cancellations (e.g. COVID-19 positivity or patient withdrawal) were equally distributed.

Previous evidence comparing tNC and AC has been inconclusive. A 2025 Cochrane review (Ghobara *et al*., 2025) found no significant differences in LBR, although data quality was low and miscarriage outcomes were underreported. In our raw data, embryo morphology differed significantly between groups, prompting the use of GLM to adjust for age, BMI, embryo stage, and endometriosis.

After adjustment, GLMs confirmed significantly higher clinical pregnancy rates in the tNC group (OR=0.37; 95% CI: 0.13-1.02; *p*=0.047) and a significantly higher miscarriage rate when AC (OR=2.96; 95% CI: 1.51-5.96; *p*=0.002). No significant differences were found in birth rates or neonatal birth weight (Table 4). These findings support the hypothesis that the physiological presence of the CL may enhance implantation and early pregnancy maintenance (Lee *et al*., 2022). Also, they contradict the lack of difference presented by Ho *et al*. (2024) in terms of clinical pregnancies or miscarriage rates when comparing AC and tNC.

Subgroup analysis of six progesterone regimens revealed that oral DYG in tNC was associated with significantly higher pregnancy rates compared to both natural-vaginal (OR=2.35; 95% CI: 1.06-5.21; *p*=0.03) and artificial-oral protocols (OR=0.87; 95% CI: 0.22-0.93; *p*=0.036). Miscarriage rates were higher in the artificial-oral subgroup compared to natural-oral (OR=1.31; 95% CI: 1.20-1.95; *p*=0.001), with no additional differences between vaginal and combined formulations (Table 5 and Supplementary Tables 1-3).

Concerns have been raised regarding the absence of the CL in AC protocols, particularly in relation to vascular and endocrine factors such as relaxin, nitric oxide, and endothelial growth factors (Lee *et al*., 2022). Although our sample was not powered to assess rare obstetric complications, we found no significant difference in hypertensive disorders between groups. Notably, gestational diabetes was significantly more frequent in the AC group (17.6%) compared to tNC (5.4%, *p*=0.03), despite the occurrence of four twin pregnancies (4.6%) in the tNC group-one of which resulted in preterm labor following feto-fetal transfusion ablation.

Although BW were slightly higher in the AC group, this difference did not achieve significance. Two congenital anomalies were reported in the AC cohort: one case of postaxial polydactyly in a MVP cycle, and one cardiac malformation following DYG exposure, which resulted in neonatal ICU admission and death from iatrogenic sepsis.

Our study is strengthened by its single-center design and uniform embryo quality, enabling control of key confounders through multivariable modelling. Limitations such as serum progesterone levels at FET were not systematically assessed, though previous studies have suggested that inadequate luteal progesterone may compromise AC outcomes. Although oocyte donation was more common in AC cycles (17/99 *vs*. 8/202), this was controlled for GLM models. Finally, C-section was frequent and largely physician directed, as common in Brazilian obstetric care.

## CONCLUSION

In this study true natural cycles were associated with significantly higher clinical pregnancy rates and lower miscarriage rates compared to artificial cycles. Subgroup analyses suggest a potential advantage for oral progesterone support in natural cycles. Our findings reinforce the physiological and clinical benefits of corpus luteum-supported endometrial preparation but prospective studies are needed to optimize luteal support strategies and assess long-term maternal and neonatal outcomes.

## Figures and Tables

**Supplementary Table 1:** Cross-analysis of Generalized Linear Models (GLM) among the subgroups

Dependent variable	Category	Estimative	p
Birth	Natural combined - artificial combined	0.76 (0.02 - 2.81)	0.298
	Natural vaginal^R^ - artificial vaginal	1.01 (0.23 - 4.76)	0.999
	Natural oral^R^ - artificial oral	1.19 (0.36 - 3.50)	0.820
Pregnancy (β-hCG +)	Natural combined^R^ - artificial combined	1.10 (0.32 - 5.89)	0.059
	Natural vaginal^R^ - artificial vaginal	0.95 (0.354 - 1.77)	0.281
	Natural oral^R^ - artificial oral	0.87 (0.22 - 0.93)	**0.036**
Clinical pregnancy	Natural combined^R^ - artificial combined	0.54 (0.04 - 6.21)	0.625
	Natural vaginal^R^ - artificial vaginal	_	_
	Natural oral^R^ - artificial oral	0.13 (0.01 - 4.13)	0.251
Abortion	Natural combined^R^ - artificial combined	_	_
	Natural vaginal^R^ - artificial vaginal	1.02 (0.22 - 4.83)	0.536
	Natural oral^R^ - artificial oral	1.31 (1.20 - 1.95)	**0.001**
Birth weigth	Natural combined^R^ - artificial combined	104.22 (-452.3 - 660.7)	0.976
	Natural vaginal^R^ - artificial vaginal	99.23 (-277.7 - 476.2)	0.936
	Natural oral^R^ - artificial oral	_109.30(-969.7-751.1)	0.992
Birth weigth (37 - 39 w)	Natural combined^R^ - artificial combined	79.59 (-488.2 - 720.8)	0.991
	Natural vaginal^R^ - artificial vaginal	52.27 (-284.5 - 378.0)	0.984
	Natural oral^R^ - artificial oral	476.75 (-268.5 - 1162.3)	0.446
Birth weigth (>39 w)	Natural combined^R^ - artificial combined	35.24 (-1005.8 - 517.2)	1.00
	Natural vaginal^R^ - artificial vaginal	249.09 (-552.0 - 999.0)	0.859
	Natural oral^R^ - artificial oral	92.98 (-896.2 - 1081.7)	0.996

Note: CI=confidence interval; *p*=statistical significance; R=reference values.

p-values are adjusted using the Holm method. All analyses were controlled for age. BMI. embryonic stage. and presence of endometriosis.

**Supplementary Table 2:** Analyses of Generalized Linear Models (GLM) among the natural preparation subgroups

Dependent variable	Category	Estimative	p
Birth	Natural combined^R^ - natural vaginal	0.88 (0.06 - 5.50)	0.356
	Natural combined^R^ - natural oral	0.92 (0.07 - 8.43)	0.439
	Natural vaginal^R^ - natural oral	1.04 (0.39 - 5.46)	0.871
Pregnancy (B-hCG+)	Natural vaginal^R^ - natural combined	0.98 (0.29 - 3.22)	0.97
	Natural vaginal^R^ - natural oral	2.35 (1.06 - 5.21)	**0.03**
	Natural oral^R^ - natural combined	0.41 (0.11 - 1.58)	0.198
Clinical pregnancy	Natural combinado® -natural vaginal	1.58 (0.16 - 15.55)	0.694
	Natural combinado® -natural oral	4.05 (0.22 - 73.87)	0.345
	Natural vaginal® -natural oral	2.56 (0.06 - 6.21)	0.401
Abortion	Natural combined^R^ - natural vaginal	_	_
	Natural combined^R^ - natural oral	_	_
	Natural vaginal^R^ - natural oral	0.489 (0.11 - 2.14)	0.377
Birth weight	Natural combined^R^ - natural vaginal	-63.62 (-487.8 - 360.6)	0.987
	Natural combined^R^ - natural oral	-300.73 (-751.7 - 150.2)	0.464
	Natural vaginal^R^ - natural oral	-237.11 (-516.9 - 42.7)	0.260
Birth weigth (37 - 39 w)	Natural combined^R^ - natural vaginal	-51.08 (-525.2 - 447.5)	0.995
	Natural combined^R^ - natural oral	-439.25 (-941.8 - 143.9)	0.288
	Natural vaginal^R^ - natural oral	-388.16 (-691.2 - -29.0)	0.061
Birth weigth (> 39 w)	Natural combined^R^ - natural vaginal	112.93 (-718.8 - 838.6)	0.980
	Natural combined^R^ - natural oral	-221.82 (-1089.3 - 517.2)	0.867
	Natural vaginal^R^ - natural oral	-334.76 (-796.7 - 104.8)	0.312

Note: CI=confidence interval; *p*=statistical significance; R=reference values.

*p*-values are adjusted using the Holm method. All analyses were controlled for age. BMI. embryonic stage. and presence of endometriosis.

**Supplementary Table 3:** Analyses of Generalized Linear Models (GLM) among the artificial preparation subgroups

Dependent variable	Category	Estimative	p
Birth	Artificial combined^R^ - artificial vaginal	1.17 (0.73 - 11.04)	0.626
	Artificial combined^R^ - artificial oral	0.95 (0.84 - 1.06)	0.994
	Artificial vaginal^R^ - artificial oral	0.89 (0.04 - 4.42)	0.904
Pregnancy (B-hCG)	Artificial vaginal^R^ - artificial combined	2.56 (0.74 - 8.80)	0.135
	Artificial vaginal^R^ - artificial oral	1.65 (0.35 - 7.76)	0.521
	Artificial oral^R^ - artificial combined	1.54 (0.27 - 8.82)	0.624
Clinical pregnancy	Artificial combined® - Artificial vaginal	_	_
	Artificial combined® - Artificial oral	1.92 (0.09 - 39.86)	0.673
	Artificial vaginal® - Artificial oral	_	_
Abortion	Artificial vaginal^R^ - artificial combined	3.41 (0.51 - 22.45)	0.201
	Artificial vaginal^R^ - artificial oral	3.40 (0.29 - 38.68)	0.323
	Artificial oral^R^ - artificial combined	1.01 (0.08 - 11.65)	0.998
Birth weight	Artificial combined^R^ - artificial vaginal	-68.62 (-587.3 - 450.0)	0.991
	Artificial combined^R^ - artificial oral	-514.26 (-1439.5 - 410.9)	0.613
	Artificial vaginal^R^ - artificial oral	-445.64 (-1360.0 - 468.7)	0.705
Birth weigth (37 - 39 w)	Artificial combined^R^ - artificial vaginal	-78.41 (-571.6 - 261.3)	0.983
	Artificial combined^R^ - artificial oral	-42.09 (-837.4 - 700.7)	0.999
	Artificial vaginal^R^ - artificial oral	36.31 (-672.5 - 752.61)	0.999
Birth weigth (> 39 w)	Artificial combined^R^ - artificial vaginal	326.79 (-724.3 - 1331.0)	0.866
	Artificial combined^R^ - artificial oral	-164.08 (-1365.9 - 1019.1)	0.987
	Artificial vaginal^R^ - artificial oral	-490.54 (-16.87 - 733.6)	0.765

Note: CI=confidence interval; *p*=statistical significance; R=reference values.

*p*-values are adjusted using the Holm method. All analyses were controlled for age. BMI. embryonic stage. and presence of endometriosis.
